# The new social landscape: Relationships among social media use, social skills, and offline friendships from age 10–18 years

**DOI:** 10.1016/j.chb.2024.108235

**Published:** 2024-04-05

**Authors:** Silje Steinsbekk, Oda Bjørklund, Patti Valkenburg, Jacqueline Nesi, Lars Wichstrøm

**Affiliations:** a Department of Psychology, Norwegian University of Science and Technology, Norway; b Amsterdam School of Communication Research, University of Amsterdam, Netherlands; c Department of Psychiatry & Human Behavior, Warren Alpert Medical School of Brown University, Providence, RI, USA; d Department of Child and Adolescent Psychiatry, St. Olavs University Hospital, Trondheim, Norway

**Keywords:** Social media, Social skills, Adolescence, Friends, Within-person, Moderators

## Abstract

Social media has created a new social landscape for adolescents. Knowledge is needed on how this landscape shapes adolescents’ social skills and time spent with friends, as these outcomes are important to mental health and psychosocial functioning. Using five waves of biennially collected data from a birth cohort assessed throughout age 10–18 years (n = 812), we found that increased social media use predicted more time with friends offline but was unrelated to future changes in social skills. Age and sex did not moderate these associations but increased social media use predicted declined social skills among those high in social anxiety symptoms. The findings suggest that social media use may neither harm nor benefit the development of social skills and may promote, rather than displace, offline interaction with friends during adolescence. However, increased social media use may pose a risk for reduced social skills in socially anxious individuals.

## 1. Introduction

Social competence refers to the ability to engage in meaningful interactions with others (Junge et al., 2020). To successfully accomplish this, children and adolescents must have a range of social skills —the building blocks of social competence (Junge et al., 2020; Rose-Krasnor, 1997). Throughout childhood and adolescence, social skills develop in interaction with the social environment, including parents, family, and friends. While not long ago, such interactions solely took place face-to-face, teens now spend an average of almost 4 h daily on social media like TikTok, Instagram, and Snapchat (Statista, 2023). However, questions remain as to how social media use affects the development of social skills, which is important to know given the beneficial outcomes of such skills and the drawbacks of lacking them in terms of mental health (Obradovic et al., 2010), academic achievement (Wentzel, 2017), coping, and self-esteem (Beauchamp & Anderson, 2010; Bijstra & Jackson, 1998).

On the one hand, it has been argued that social media interactions provide fewer affordances to practice social skills, thus negatively impacting the development of such skills. On the other hand, it has been hypothesized that social media may constitute an additional arena for social interaction, potentially promoting social skills via more opportunities to interact. This study is the first to examine the validity of these contrasting hypotheses. In addition, it investigates whether increased social media use predicts changes in time spent with offline friends. In doing so, we investigate another set of competing hypotheses: the displacement hypothesis (Kraut et al., 1998), which states that online activities leave less time for offline activities, and the compensation hypothesis (Hooghe & Oser, 2015), which argues that online interactions complement or may even enhance offline interactions for some individuals (Kraut et al., 2002). Because face-to-face interactions with friends are related to increased happiness (Quoidbach et al., 2019), a sense of belonging (Chopik, 2017), and less loneliness (Achterhof et al., 2022), such knowledge is needed.

Although peer interactions can take place both offline and online, the two settings may provide different opportunities for developing social skills. Offline peer interactions are regarded as fundamental to human sociality (Hamilton & Holler, 2023). It has been shown, for example, that imitation of facial emotional expressions is more likely to appear in face-to-face settings (Hirsch et al., 2023), and such mimicry is related to the ability to process social information (Genschow et al., 2018). In many cases, online interactions lack the ‘richness’ of offline interactions (Nesi et al., 2018a; Nesi et al., 2018a) and may therefore be less likely to nurture social skills. Further, because online interactions provide fewer nonverbal cues than offline ones do (Lieberman & Schroeder, 2020; Nesi et al., 2018b), the risk of misunderstandings and communication difficulties may increase, potentially harming the development of social skills. Finally, the displacement hypothesis suggests social media use to negatively impact time spent with offline friends.

However, social media also represents an additional interpersonal arena (Nesi et al., 2018a) that may add to the offline one. This could offer adolescents increased possibilities for practicing and thus *enhancing* social skills and engagement with friends, which may *facilitate* face-to-face interaction. It has indeed been shown that online peer communications may promote adolescents’ sense of belonging (Davis, 2012), provide opportunities for forming new social ties and strengthen weak ones (Lieberman & Schroeder, 2020), and are positively associated with social capital and improved relationship quality (Appel et al., 2020; Desjarlais & Joseph, 2017; Dredge & Schreurs, 2020). Because offline friends often also are online friends (Reich et al., 2012; van Zalk et al., 2014), online interactions may, therefore, motivate youth to spend more time with friends offline rather than make them devote less time to offline friends, a prediction we hereby test.

Existing research has only examined a few of the many social skills that are important for individuals’ social competence (Cheung et al., 2017; Junge et al., 2020), and primarily, social skills have been assessed as *predictors* of social media use (e.g., empathy and perspective taking Bowman-Smith et al., 2021; Riley et al., 2022), rather than as outcomes. Some notable exceptions exist, including two studies reporting social media use to predict more empathy in adolescents one year later (Lozada & Tynes, 2017; Vossen & Valkenburg, 2016). There is an urgent need to assess the association of social media use with a broader range of social skills, as the sum of skills is likely more important for how effectively an individual interacts compared to the impact of single skills. Furthermore, summarizing a handful of small-scale studies of adults, Hall and Liu (2022) noted that days with more social media use may involve fewer face-to-face interactions, but whether social media use predicts a decline in offline time spent with friends during the adolescent years is yet to be explored.

Examining how the development of skills and time spent with offline friends is predicted by social media use is particularly important across childhood and adolescence, a sensitive period of social development (Orben et al., 2020). Further, there is a need to examine the relationship between social media use and social outcomes at the level of the individual. Until recently, scholars have mainly captured a blend of within and between-person effects (Pouwels et al., 2021, 2022), although media effects occur solely *within* persons (Potter, 2011; Valkenburg & Peter, 2013). More specifically, little is known about whether changes in an individual’s level of social media use predict changes in that individual’s social skills or time spent with friends offline, compared to what it otherwise would be (i.e., using him/herself as the control), not compared to others. We therefore add to existing research by separating within-from between-person effects when examining the relation between social media use and social skills and time spent with offline friends, respectively, using data from a Norwegian birth cohort of children assessed biennially from age 10–18 years.

Finally, whether social media use promotes or impairs social skills—and displaces or reinforces time spent with friends offline—might depend on characteristics of the individual and his/her social media use. For some individuals, using social media may confer negative social outcomes, whereas for others the impact might be positive, as indicated by the Differential Susceptibility to Media Effects Model (DSMM) (Valkenburg & Peter, 2013). According to this model, three types of differential-susceptibility variables may moderate relations between social media use and outcomes: 1) dispositional, 2) developmental, and 3) social variables (Valkenburg & Peter, 2013). In the present work, we test moderators within each of these three categories.

As for dispositional variables, we focus on sex and social anxiety. First, because girls are more likely to use social media for communicative purposes (Garrett et al., 2023; Pew Research Center, 2022), have more intimate friendships, disclose more (Rose & Asher, 2017; Rose & Rudolph, 2006), and often start their developmental trajectories of social skills at an earlier stage than boys (Feraco & Meneghetti, 2023; Hajovsky et al., 2022), the impact of social media use on social development may differ between the sexes. We therefore examine if sex, as a dispositional variable, moderates the relationship between social media use and the two outcomes in question.

Second, youth with social anxiety symptoms have been shown to benefit more from social media use, yet also be more vulnerable to, social media use. To exemplify, one study found online self-disclosure to be positively related to social connectedness and well-being for those high in social anxiety, whereas a negative relation was found for those displaying low levels of anxiety symptoms (Desjarlais, 2022). Another study found perceived cue absence and asynchronicity to enhance online communication and emotional processes in socially anxious youth, whereas the opposite was true for non-anxious individuals (Angelini & Gini, 2023). On the other hand, social anxiety is linked to more problematic social media use (e.g., greater frequency and intensity, addiction) (O’Day & Heimberg, 2021). These opposing findings might be viewed in light of the social compensation/poor-get-richer and social enhancement/rich-get-richer (i.e., poor-get-poorer) hypotheses, respectively. As suggested by the former, characteristics of social media (e.g., non-physical presence of others) might compensate for features of offline conversations that socially anxious individuals find difficult, and thus make them use, - and benefit more from social media. As indicated by the latter hypothesis though, socially anxious individuals’ use of social media may enhance their difficulties (e.g., heightened need for assurance) increasing the risk for problematic use (Lee-Won et al., 2015). In the present inquiry, we therefore include symptoms of social anxiety as a potential dispositional moderator between social media use and the outcomes in question.

As for developmental variables, we focused on age. Frequency of social media use increases by age (Common Sense, 2022), and its impact on social skills development and time spent with friends may change accordingly. We hence investigate whether the predictions to and from social media use differ by age, which constitutes the developmental differential-susceptibility variable included. Lastly, as for social variables, we investigated the moderating influence of friendship closeness. Social media use has been linked to both higher (Nesi et al., 2018a; Uhls et al., 2017; Valkenburg & Peter, 2011; Yau & Reich, 2018) and lower closeness to friends (Sbarra et al., 2019), which likely applies to different individuals (Pouwels et al., 2021). It may be that those who experience high closeness to friends have less to gain from the extra opportunity that social media may present in terms of self-disclosure and support (Desjarlais & Joseph, 2017), but they may also be better positioned to gain closeness online, which may promote social skills (Glick & Rose, 2011) and protect against a potential decline in time spent with offline friends. We test if closeness to friends, as a social differential-susceptibility moderator, impacts the relation between social media use and the two outcomes in question.

## 2. Methods

### 2.1. Design and participants

This preregistered study uses data from the Trondheim Early Secure Study (TESS). All children born in Trondheim in 2003 and 2004 (N = 3456) were invited to participate in the year they were 4. An invitation letter together with The Strengths and Difficulties Questionnaire (SDQ) version 4–16 (Goodman et al., 2000), a screening assessment for emotional and behavioral problems, was sent to their parents by mail. Parents brought the completed SDQ when attending the local health care clinic for their offspring’s mandatory 4-year-old health check-up (N = 3358), where a health nurse informed them about the study. The health nurses missed asking 166 parents and 176 were excluded due to inadequate language proficiency. In total, 2477 parents signed the consent form granting permission for their children to participate, but as detailed below, only a proportion of those consenting were selected into the main study.

Because the initial, overarching aim of TESS was to study mental health, we oversampled for children with emotional and behavioral problems, thus increasing variance and statistical power. More specifically, children were allocated to four strata according to their SDQ scores (cut-offs: 0–4, 5–8, 9–11, and 12–40), and the probability of selection increased with increasing SDQ scores (0.37, 0.48, 0.70, and 0.89 in the four strata, respectively (i.e., the higher SDQ scores, the higher odds for being drawn to the study)). This oversampling was corrected for in the analyses. In total, 1250 of those who consented were drawn to participate. From age 4 onwards (N = 1007) participants have been thoroughly assessed at the university clinic every second year, with 8 data waves completed, including information from the participant’s parents and teachers. Procedure and flow of participants are displayed in Fig. 1.

Social media use was measured from age 10 onwards; thus the present study only captures the last five waves (M_age_ 10 (2013/2014, n = 704, 52.1% girls, 47.9% boys, M_age_ = 10.51, SD = 0.17), 12 (2015/2016, n = 666, 51.7 % girls, 48.3% boys, M_age_ = 12.49, SD = 0.15), 14 (2017/2018,n = 635, 52.9 % girls, 47.1 % boys, M_age_ = 14.35, SD = 0.16), and 16 (2019/2020, n = 666, 55.0 % girls, 44,9% boys, M_age_ = 16.98, SD = 0.31) and 18 (2022/2023, n = 606, 56 % girls, 44% boys, M_age_ = 18.6, SD = 0.24). As requested by the ethical approval from the Regional Committee for Medical Research Ethics Central Norway, the children were specifically informed about the study at age 12 (parents had consented on their behalf at baseline/age 4). Further, in accordance with Norwegian law, participants provided their own written consent at age 16, when parents are no longer legally positioned to consent on their children’s behalf.

### 2.2. Measures

#### 2.2.1. Social media use

Social media use was assessed by semi-structured interviews conducted by the same trained personnel at all measurement points. Participants were asked about platforms used, overall frequency of use, and specific social media behavior. The main outcome constitutes the monthly sum of liking, commenting, and posting, which captured the participants’ responses to the following questions: 1)’How often do you like other’s updates?’; 2) ‘How often do you write comments to other’s updates or photos?’; 3) ‘How often do you post (written) updates on your own social media sites?’; 4) ‘How often do you post photos’? At ages 16 and 18, we also asked 5) ‘How often do you post selfies?’. The questions were not specific to certain social media platforms, but as the participants were interviewed, the interviewers would provide examples of social media sites if needed, or in other ways facilitate a correct recall (e. g., ‘If you think about last week … ‘).

We also validated our main analysis and tested whether the results were replicated when using an alternative means of measuring the frequency of social media use, captured by interview at ages 10, 12, and 14 (total frequency of checking social media per day) and objectively measured at ages 16 and 18 (daily time spent on social media apps according to the phone’s screen time application). For details, see Online material (Sensitivity analyses).

#### 2.2.2. Social skills

We assessed common social skills with the Social Skills Rating System (SSRS-P) (Gresham & Elliot, 1990) (ages 10, 16, 18) and the Social Skills Improvement System Rating Scales (SSIS-RS) (Gresham & Elliott, 2007) (ages 12 and 14), respectively. The latter is a revision of the former and includes 6–7 items per subscale, as opposed to 10 in the SSRS-P. For both measures, parents indicated the frequency with which their child exhibited each social skill on a 4-point scale (0 = *never;* 1 = *seldom;* 2 = *often;* 3 = *almost always*). A total social skills score was estimated based on the subscales Cooperation, Assertion, Self-control, and Responsibility.

Reliability estimates have proved to be consistent across the SSRS-P and the SSIS-RS, indicating that the subscales measure the same construct (Gresham et al., 2011). Both instruments have strong psychometric properties in terms of internal consistency and test-retest reliability (Gresham & Elliot, 1990; Merrell et al., 2001), and the Cronbach’s alphas of the total score used here ranged from α = 0.91–0.93.

#### 2.2.3. Time spent with friends offline

At all timepoints, participants responded to the following two questions: ‘Think back on the last week/7 days. How many of these days have you’: 1)’Been together with friends at their place or your place?’; 2)’Spent most of the afternoons/evenings out together with friends’? The scores on these items were summed so that higher scores indicate more days spent with friends.

#### 2.2.4. Sex

Sex was coded based on the participant’s national ID number which is based on sex assigned at birth.

#### 2.2.5. Social anxiety

To assess symptoms of social anxiety, parents and children were interviewed separately using the Child and Adolescent Psychiatric Assessment (CAPA) (Angold & Costello, 2000) at ages 10, 12 and 14 years. CAPA is based on the diagnostic criteria of the Diagnostic and Statistical Manual of Mental Disorders (DSM-IV) and interviewers posed mandatory and optional follow-up questions until they had enough information to decide whether a social anxiety symptom was present or not, if reported by either child or parent. At age 16, symptoms of social anxiety were assessed by the Norwegian version of the Schedule for Affective Disorders and Schizophrenia for School-Aged Children (K-SADS) (Kaufman et al., 1997), and as in former waves, participants and parents were separately interviewed. K-SADS is based on DSM-5, but social anxiety contains the same two symptoms in both editions of the DSM, and here we applied counts of the number of symptoms of social anxiety. Raters blind to all information recoded 77 of the CAPA videotapes and 114 of the K-SADS audiotapes, revealing an inter-rater reliability of ICC = 0.78 for the former and 0.96 for the latter.

#### 2.2.6. Friendship closeness

At all timepoints, the self-reported Network of Relationships Inventory (NRI) (Furman & Buhrmester, 1985) was used to assess relationship to best friend. As in prior work (Burk & Laursen, 2005; Chow et al., 2013; De Goede et al., 2009), we used 6 items, forming a closeness subscale (also named friendship positivity) (Burk & Laursen, 2005), constructed by the 3 items of the Emotional Support (e.g., ‘How much do you seek out this person when you’re upset?’) and Satisfaction (e.g., ‘How satisfied are you with your relationship with this person?’) subscales, respectively. Each item was rated along a five-point Likert scale (1 = almost never/not at all/very bad; 5 = almost always/very much/very good). The closeness scale displayed good internal consistency across the five measurement points (α = 0.76–0.80).

### 2.3. Statistical analyses

Analyses were conducted in Mplus version 8.3 (Muthén & Muthén, 2017), using a full information maximum likelihood procedure to handle missing data and probability weights to account for the oversampling described above using a sandwich estimator (see Methods). To test if social media use predicted the development of social skills and time spent with friends offline, respectively, two Random Intercept Cross-lagged panel models (Hamaker et al., 2015) were fitted. We created latent random intercepts for each of the study variables, which captures the average level of social media use, social skills, and time spent with friends from age 10 to 18. A latent variable was then estimated for each variable at all time points with the error variance in the observed variable set to 0, thereby transferring the variance to its latent variable. These latent variables capture the participant’s deviation from her or his own mean level during the observational period. In each of the two main models, the outcome (i.e., social skills and time spent with friends, respectively) at each time point was regressed on social media use and the outcomes two years prior.

Notably, to obtain the most parsimonious model and because we also aimed to test age effects (i.e., testing age effects will contribute to provide the most parsimonious model), we continued estimating models until the most parsimonious model for each of the two outcomes was identified. If the Satorra-Bentler scaled chi-square test (Satorra & Bentler, 2001) shows that there is no significant difference in model fit between a freely estimated model and a model where cross-lagged paths are set to be equal across time, no age effect is evident (Mulder & Hamaker, 2021). For parsimony reasons, such constrained model would be preferred, and therefore constitute the vantage point for our second step of analyses: Testing of interaction effects. Starting with sex as a moderator, multiple-group analyses were conducted (Mulder & Hamaker, 2021), comparing a model where the cross-lagged within--person coefficients (e.g., paths from social media use to social skills) were freely estimated with a model where they were identical for girls and boys, yet again using the Satorra-Bentler scaled chi-square test (Satorra & Bentler, 2001).

Because the remaining two moderators (symptoms of social anxiety and closeness to friends) are continuous variables, the potential impact of these was examined by adding an interaction term to the main RI-CLPM models (Speyer et al., 2023). In these models, interactions were composed of the within-person centered predictor (social media use) and the observed time-varying moderator (Speyer et al., 2023) (symptoms of anxiety and friendship closeness, respectively), thus capturing the interaction between changes in social media use and the individual’s overall level of the moderator in question.

Finally, applying the same within-person approach as described above, we conducted sensitivity analyses testing whether changes in an alternative measure of social media use frequency (i.e., overall checking/time spent on social media) predicted changes in social skills and time spent with friends, respectively (Online material). For all analyses, two-sided p-values <.05 were regarded as statistically significant.

## 3. Results

Descriptive statistics are presented in Table 1. As displayed, the mean frequency of liking, commenting, and posting steadily increased from age 10 to 12 and 14 years before stabilizing and then declining from age 16 to 18. No cross-sectional associations between social media use and social skills were found, whereas more time spent with offline friends was concurrently associated with greater social media use at ages 10, 12, and 14 years.

The freely estimated models displayed good fit (Social media use and social skills: χ^2^ = 29.558; CFI = 0.994; TLI = 0.987; RMSEA = 0.022 (90% CI: 0.00, 0.040); SRMR = 0.054.; Social media use and time spent with friends: χ^2^ = 27.633; CFI = 0.987; TLI = 0.972; RMSEA = 0.020 (90% CI: 0.00, 0.038); SRMR = 0.032). These models are depicted in the Online material (Fig. S1 and Fig. S2) and show that no significant within-person cross-lagged relations exist, except that increased social media use predicts more time with friends from age 12–14 years.

As noted above, to test whether the cross-lagged paths differed by age we examined whether freely estimated models would have a better fit to the data than models where the cross-lagged paths were set to be equal across time. Results revealed that for both outcomes, they did not fit better (social media use and social skills: Δdf = 6; Δχ2 = 4.58; *p* = 0.60; social media use and time spent with friends: Δdf = 6; Δχ2 = 7.43; *p* = 0.28); thus, no age effects were evident. These constrained—and thus most parsimonious—models thus constitute the final ones. The results of these models are displayed in Fig. 1, showing good fit (social media use and social skills: χ2 = 34.22; CFI = 0.995; TLI = 0.991; RMSEA = 0.018 (90%CI: 0.00, 0.035); SRMR = 0.055; social media use and time spent with friends: χ2 = 35.073; CFI = 0.984; TLI = 0.974; RMSEA = 0.019 (90%CI: 0.00, 0.035); SRMR = 0.036.). Note that constraining the paths to be equal across time only indicates that the unstandardized cross-lagged estimates are invariant and thus identical (as displayed in Fig. 1); the standardized lagged effects may still differ slightly over time (Mulder & Hamaker, 2021).

As shown, increased use of social media was unrelated to future levels of social skills across ages 10–18 years (Fig. 1a), but positively predicted time spent with friends offline (Fig. 1b). Thus, participants who liked, commented, and posted more over time displayed an increase in the number of days they spent with friends offline. The mean of the four standardized coefficients (Ages 10–12, 12–14, 14–16, 16–18 years, respectively) shows that the effect was small (mean *β* across ages 10–18 = 0.10). As regards the opposite direction of influence, time spent with friends or social skills did not predict future social media use. All estimates including 95% CI’s and *p*-values are displayed in Table S1 (Online material).

Multi-group analyses according to sex showed that within-person relations between social media use and the two outcomes examined did not differ between boys and girls (social media use and social skills: Δdf = 2; Δχ2 = 3.15; *p* = 0.21; social media use and time spent with friends: Δdf = 1; Δχ2 = 0.71; *p* = 0.40).

As a final step of the main analyses, we tested whether symptoms of social anxiety and friendship closeness moderated the cross-lagged relations (Table 2; Descriptives are displayed in Table S2, Online material). Because the model did not converge when examining all interaction terms in one model, each interaction was added separately such that each model only included one interaction term predicting the outcome at one time point (e.g., social media x social anxiety at T4 predicting social skills at T5). From age 12 to 14 and 14–16 years, the interaction term between social media use and the intercept (i.e., overall level) of social anxiety was negative and significant, indicating that increased social media use forecasted a small decline in social skills among those with higher levels of social anxiety symptoms (12–14 years: B = − 0.11, 95% CI = − 0.19, − 0.02, *p* = 0.016, β = − 0.12; 14–16 years: B = − 0.07, 95% CI = − 0.15, − 0.00, *p* = 0.040, β = − 0.06), and from age 16 to 18 only, this also applied to those reporting more friendship closeness (B = − 0.09, 95% CI = − 0.18, − 0.00), *p* = 0.040, β = − 0.19). No moderation effects were evident for the time spent with offline friends as the outcome.

Results of the sensitivity analyses confirmed the main findings (Online material, Table S3 and Table S4). No relationships between social media use and social skills were found, but youth who increased their overall frequency of social media use spent more time with offline friends across ages 10–18 years. Also, the sensitivity analyses showed those who spent more days with friends across the measurement period displayed increased social media use over time (i.e., a reciprocal relation).

## 4. Discussion

During the transition from childhood to adolescence, socializing with peers outside of the family is central to socioemotional development and well-being. Thus, as young people have increasingly turned to social media for interpersonal interactions, concerns have been raised that social media use may displace time adolescents spend with friends offline and may negatively impact the development of social skills. Using five waves of biennially collected data from a Norwegian birth cohort of children, separating within- and between-person effects, we found no support for the assumption that social media use predicts declines in social skills. Our findings further showed that increased social media use was predictive, although not strongly, to increased time spent with friends offline. Youth who liked, commented, and posted more on social media at one time point relative to their overall level of such social media use, spent more days with friends at the next assessment point, compared to what they otherwise would do. This prediction was evident across ages 10–18 years. Neither sex nor age affected the relationship between social media use and the two outcomes in question but increased social media use predicted declined social skills in youth with more social anxiety symptoms (from age 12 to 14 and 14–16 years), though effects were small (β = − 0.12; β = − 0.06, respectively).

Prior theoretical work suggests that social media use could either promote or hamper the development of social skills, but we found no support for either effect. Former research has shown that online chatting is associated with improved offline perspective-taking (Alloway et al., 2014) and increased empathy (Lozada & Tynes, 2017; Vossen & Valkenburg, 2016), which are important components of social skills, thus contrasting the current results. Notably, these studies did not separate within-from between-person effects and addressed specific, rather than overall, social skills, which might explain the diverging findings. More importantly, we examined the frequency of liking, commenting, and posting, which are characterized as masspersonal social media use (i.e., one-to-many) whereas chatting, assessed in the above studies, mostly pertains to dyadic interactions (i.e., one-to-one) (Utz, 2015). Dyadic online interactions are more closely related to self-disclosure and connectedness (Desjarlais, 2022; Utz, 2015), potentially elucidating the reason behind their positive impact on offline social skills. Desjarlais (2022) found that masspersonal computer-mediated communication predicted connectedness due to online self-disclosure in socially anxious individuals, thus supporting the poor-get-richer hypothesis. Our findings, on the other hand, align with prior research supporting a poor-get-poorer (i.e., rich-get-richer) hypothesis, as we found socially anxious individuals to have an increased risk for declined social skills if they increase their social media use (Bowden-Green et al., 2020; O’Day & Heimberg, 2021). A meta-analysis testing these competing hypotheses also found no support for the poor-get-richer hypothesis, as socially anxious individuals did not get socially ‘*richer*’ using social media, although they made greater *use* of social media (i.e., compensation-hypothesis) (Cheng et al., 2019). The authors conclude that the relations are complex and the hypotheses likely valid under different conditions, noting that a distinction should be made between social media use and benefit of such use (Cheng et al., 2019).

Importantly, the interaction effect found in the present study was small, thus the finding should be interpreted with caution. However, if we were to speculate on why socially anxious individuals might experience impaired social skills due to increased social media use, attention could be directed towards upward social comparison. This is a central mechanism linking social media use to negative outcomes (e.g., body image) (McComb et al., 2023), and may particularly apply to those who are socially anxious due to their maladaptive cognitive patterns (e.g., rumination, self-focused attention, negative self-evaluations) (Chiu et al., 2021). To exemplify, socially anxious adolescents have fewer friends and are less likely to report friendship closeness and social acceptance than those low in social anxiety (La Greca & Lopez, 1998). Being exposed to the idealized versions of other’s social lives on social media may therefore create a greater gap between this ‘reality’ and how the socially anxious youth perceive her/his own social life, compared to youth who are not socially anxious. Such social comparison may reinforce the socially anxious person’s view of her/himself (i.e., as socially insecure) and his/her social difficulties (e.g., social withdrawal), potentially causing declined social skills.

Surprisingly, our results also showed that increased social media use was associated with declined social skills in those reporting higher friendship closeness, but only from age 16–18 years (β = − 0.19). Although we see no highly reasonable explanations for such moderation effect only in late adolescence, one could point to that late adolescents have fewer friends and more intimate friends than at earlier ages (De Goede et al., 2009; LaFontana & Cillessen, 2010; Neal, 2023). Being engaged in less, although closer friendships may reduce opportunities to develop social competence in various relationships and across settings (Barzeva et al., 2022), possibly explaining the current decline in social skills. Nevertheless, future studies should replicate this rather odd finding and examine the potential mechanisms involved.

Participants who increased their social media use showed a small increase in the number of days spent with friends offline. Studies of adults also report that digital interaction reinforces rather than displaces offline contact over time (Danielsbacka et al., 2023; Stevic et al., 2021), although null findings are just as evident (Hall et al., 2019; Hall & Liu, 2022). To the best of our knowledge, the present study is the first to examine the relation between social media use and time spent with offline friends at the within-person level and capturing the years from late childhood to emerging adulthood. Importantly, during adolescence the boundaries between offline and online peer interactions are blurred, with offline friends also being online friends (van Zalk et al., 2020) being the new norm.

Our results align with studies showing that connecting with others and maintaining relationships are important motivations for adolescents’ use of social media (Ellison et al., 2007; Kircaburun et al., 2020; Park et al., 2009), connecting with people known from offline contexts being of particular importance (Reich et al., 2012). Use of online resources is found to reinforce already existing friendships (Desjarlais & Willoughby, 2010), which may explain why social media use promotes more time spent with friends face-to-face. Although one hypothesized mechanism for the association between social media use and time spent with friends is increased closeness with friends, potentially due to more self-disclosure, neither friendship closeness nor social anxiety moderated effects in the current study. However, it should be noted that we assessed closeness to best friend, whereas the outcome measure (i.e., time spent with friends face-to-face) did not differentiate between best friend and other friends, possibly contributing to the null finding.

Online interactions not only fuel existing relationships, but also enhance the initiation of new ones (Koutamanis et al., 2013), with more than half of US adolescents having made new friends online (Lenhart, 2015). Thus, it might also be that the relationship between increased social media use and time spent with friends is partly due to new friendships. Triadic structures of conjoined friendships may be of particular importance in this regard, with youth becoming friends with members of their friends’ social networks (van Zalk et al., 2014, 2020). The present findings cannot speak to whether any of the above explanations are at play, but we hope future research will examine these potential mechanisms.

The results of our sensitivity analyses confirmed the main findings. Replacing liking, commenting, and posting with an alternative frequency of social media use revealed even slightly stronger effects, which indicates that the prediction by social media use of time spent with friends offline is not restricted to the specific social media behavior captured in the main analyses. The result of the sensitivity analyses further indicated a bidirectional relationship between social media use and face-to-face interaction with friends. Youth who spent more time with friends over time displayed increased overall social media use. The corresponding estimates of the main analyses were also positive, but non-significant. Because both the predictor and the outcome were self-reported (i.e., questionnaire and interview-based, respectively), one might assume that a reporter-bias exists, e.g., that very sociable individuals and/or those who are more conforming (i.e., being with friends is culturally attractive) overrate their social media use and number of days spent with friends offline. This could still not explain why *increased* levels of social media use or time spent with friends predict *increased* levels of the outcome in question. Further, such reporter bias is likely relatively time-invariant and thus accounted for in the analyses. Nevertheless, the present sensitivity findings should be replicated before conclusions can be drawn, especially since two different measures of overall frequency of social media use was applied in these analyses.

### 4.1. Strengths and limitations

Strengths of the present inquiry constitutes the longitudinal design, the number of follow-ups and the separation of within, - from between-person effects, in addition to the use of different informants (i.e., parent-reported social skills, self-reported social media use and time with friends). The most notable limitation is that the frequency of social media use was subjectively assessed, which only moderately correlates with device-logged, objectively measured use (Parry et al., 2021). Importantly though, we used interviews vs. questionnaires, and interviewers asked questions to facilitate correct recall, which likely improves the validity of the report. Also, we assessed social media behavior (i.e., liking, commenting, posting) that currently cannot be assessed by existing technology (e.g., the screen-time app). Another limitation of social media use research in general, and longitudinal studies such as the present in particular, is the fast-developing technology. New apps are constantly introduced, and although some features are the same (e.g., likes, commenting), there are indeed differences (e.g., text messages and still photos are not facilitated by TikTok), which may affect the impact of social media use. Importantly, the first wave of data (age 10) applied in the present inquiry was collected from November 2013 to August 2015. The number and characteristics of social media applications have substantially developed since then, thus we cannot know whether the present findings would be replicated if data collection started today or a couple of years ago. Further, even though our sample is relatively large, the null finding regarding social media use and social skills should be replicated in studies with more statistical power before definitive conclusions can be drawn. We would also like to note that although we relied on a theoretical framework in selecting moderators, other moderators not examined here might affect the relationship between social media use and the outcomes examined. As already noted, our study was also not very well powered to detect sex differences. Thus, the non-existent sex difference revealed should be replicated before conclusions can be drawn. Finally, each interaction was added separately to allow for model convergence (i.e., each model only included one interaction term predicting the outcome at one time point), thus we cannot know whether the interaction effects revealed would be significant if tested simultaneously.

### 4.2. Conclusions

In conclusion, aiming to determine if social media use predicts youth’s sociability, we tested two opposing assumptions, namely whether increased social media use (i.e., frequency of liking, commenting, and posting) at one time point predicted impaired or improved social skills and spending more or less time with friends face-to-face at the next time point, using biennially collected data from age 10–18 years. We found no support for changes in social media use predicting changes in social skills, but predicted spending more time with friends offline, although with small effects. These predictions were of similar magnitude for boys and girls and across ages. In spite of no prediction from social media use to social skills among adolescents in general, socially anxious youth who increased their social media use evinced impaired social skills later on. Such interaction effects need to be replicated before conclusions can be drawn. Our findings highlight the complex interplay of social media use and interpersonal experiences among adolescents and provide preliminary evidence that concern over declining social skills as a result of social media use may be unwarranted. Social media use may even support offline interaction with friends, and thus indirectly promote adolescents’ wellbeing and functioning.

## Supplementary Material

Appendix A. Supplementary Data

## Figures and Tables

**Fig. 1.**
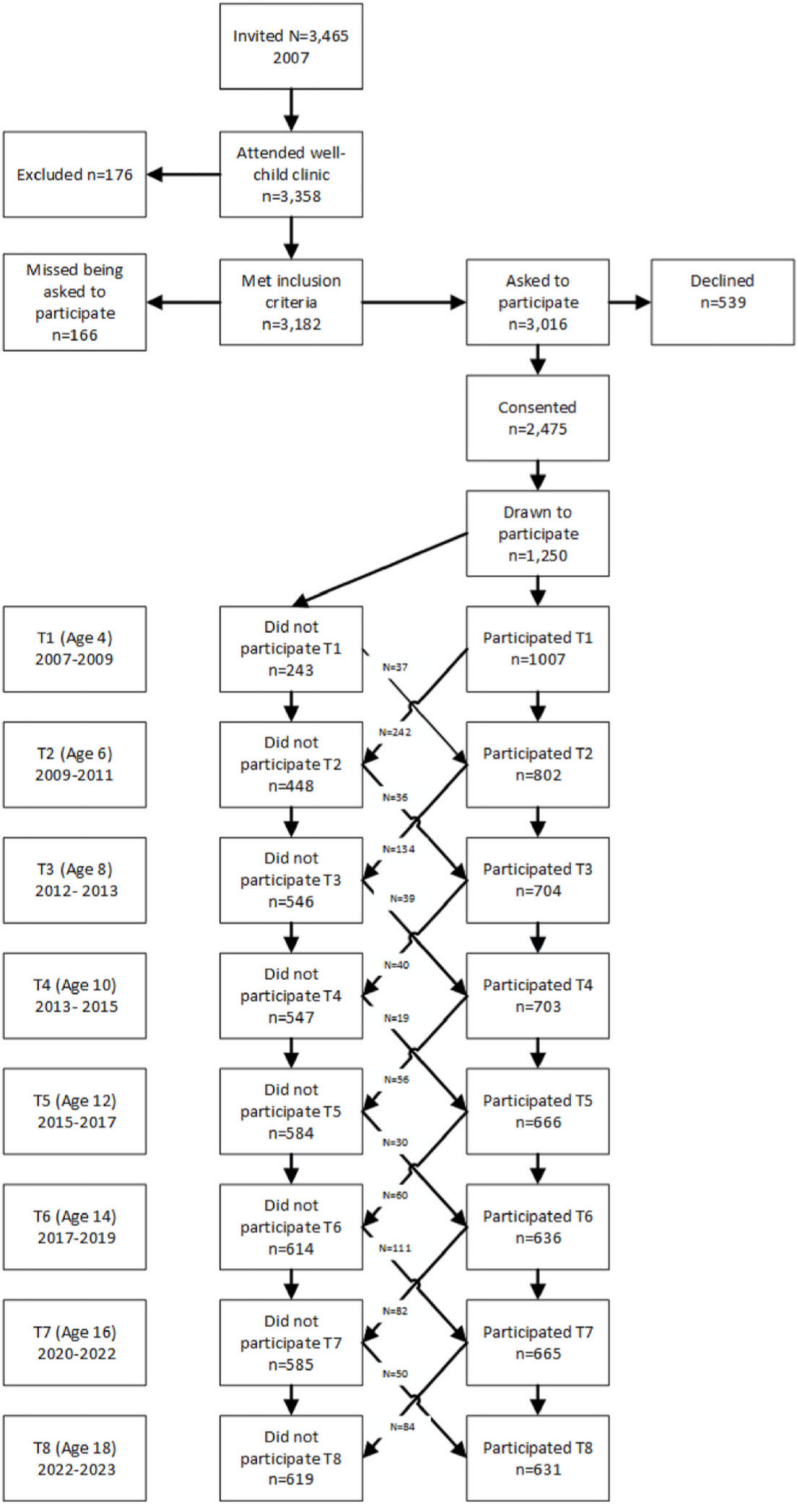
The Trondheim Early Secure Study (TESS). Procedure and flow of participants. *Note*: T1 = Time 1, T2 = Time 2 etc.

**Fig. 1a.**
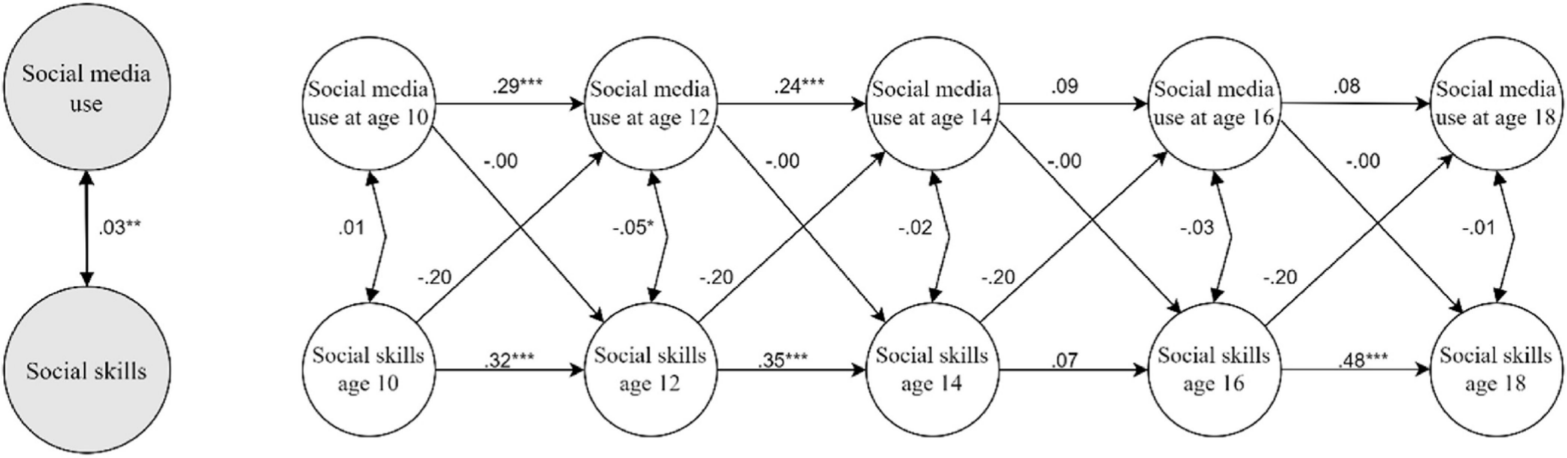
Social media use and social skills. Results from the final estimated Random Intercept Cross-lagged panel model.

**Fig. 1b.**
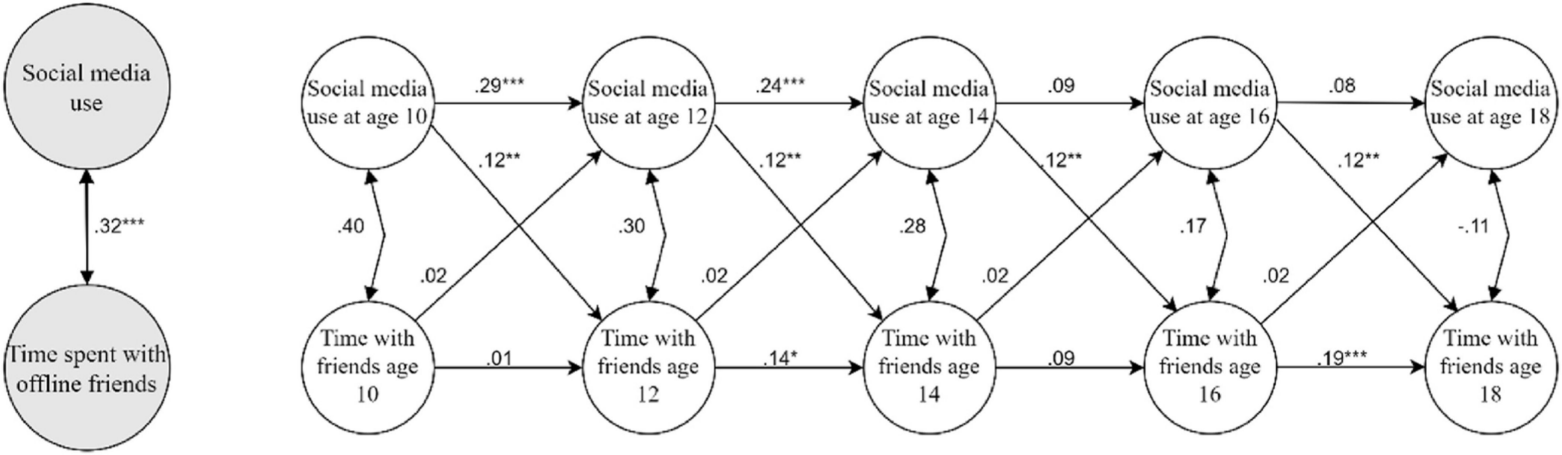
Social media use and time spent with offline friends. Results from the final estimated Random Intercept Cross-lagged panel model. *Note*. The figures display the relations between random intercept estimates (bigger grey circles) and within-person estimates (smaller white circles) of social media use and social skills (Fig. 1a), and time spent with offline friends (Fig. 1b), respectively (i.e., two separate models). Unstandardized estimates are displayed. For presentation purposes, only the latent variables are displayed, not the observed study variables on which they are based. As detailed in the Results section, cross-lagged paths were constrained, resulting in identical estimates across time points, whereas autoregressions are freely estimated and thus vary.

**Table 1:** Descriptive statistics and bivariate correlations between main study variables.

Variables	Mean (SD)	Min/max	1	2	3	4	5	6	7	8	9	10	11	12	13	14	15

1. Social media use at age 10	27.73 (44.18)	0.0/400.0	-														
2. Social media use at age 12	59.61 (83.72)	0.0/1139.0	0.17***	-													
3. Social media use at age 14	78.96 (54.38)	0.0/630.0	0.16***	0.30***	-												
4. Social media use at age 16	75.90 (276.76)	1.0/3177.0	0.16	0.07	0.05	-											
5. Social media use at age 18	25.20 (123.66)	2.0/1807.0	0.02	0.04*	− 0.01	− 0.02	-										
6. Social skills at age 10	3.01 (0.35)	1.78/3.88	0.05	0.05	0.11**	0.04	0.01	-									
7. Social skills at age 12	3.30 (0.35)	2.18/4.00	− 0.03	− 0.09*	0.01	0.07	− 0.02	0.68***	-								
8. Social skills at age 14	3.31 (0.34)	1.94/4.00	0.04	− 0.04	0.03	0.03	0.04	0.64***	0 74***	-							
9. Social skills at age 16	3.24 (0.33)	2.20/3.93	0.02	0.02	0.06	0.002	0.01	0.52***	0.57***	0.62***	-						
10. Social skills at age 18	3.27 (0.34)	1.78/3.93	− 0.02	− 0.02	− 0.002	0.01	− 0.04	0.50***	0.53***	0.60***	0.75***	-					
11. Time spent with friends at age 10	3.29 (2.71)	0.0/14.0	0.12*	0.04	0.16**	0.05	0.05	0.02	0.003	0.04	0.09*	0.03	-				
12. Time spent with friends at age 12	3.83 (2.95)	0.0/14.0	0.13**	0.11**	0.15**	− 0.05	0.02	0.04	− 0.06	− 0.002	− 0.01	0.002	0.25***	-			
13. Time spent with friends at age 14	3.86 (2.93)	0.0/14.0	0.06	0.09*	0.27***	0.05	0.01	0.08	− 0.001	0.004	− 0.004	− 0.002	0.20***	0.34***	-		
14. Time spent with friends at age 16	4.17 (3.18)	0.0/14.0	0.05	0.08	0. 17***	0.02	− 0.01	0.04	− 0.03	0.002	0.06	0.07	0.20***	0.25***	0.27***	-	
15. Time spent with friends at age 18	4.37 (3.06)	0.0/14.0	0.08	0.05	0.16**	− 0.03	− 0.02	− 0.02	− 0.06	− 0.07	− 0.05	− 0.03	0.26***	0.30***	0.24***	0.38***	-

Note.

* p < 0.05

** p < 0.01

*** p < 0.001. Social media use = Frequency of monthly liking, commenting, and posting.

**Table 2:** Symptoms of social anxiety and friendship closeness as moderators of the relations between social media use and time spent with offline friends.

	B	SE	95% CI	β	p value	B	SE	95% CI	β	p value
		
	Social skills at age 12					Time spent with friends at age 12				

SoMe X social anxiety age 10	− 0.05	0.05	− 0.15, 0.05	− 0.05	0.33	− 0.10	0.89	− 1.86, 1.65	− 0.01	0.91
SoMe X friendship closeness age 10	0.04	0.02	− 0.002, 0.09	0.12	0.06	− 0.04	0.21	− 0.44, 0.37	− 0.01	0.85
	Social skills at age 14					Time spent with friends at age 14				
		
SoMe X social anxiety age 12	− 0.11	0.05	− 0.19, − 0.02	− 0.12	0.02	0.21	0.38	− 0.53, 0.95	0.02	0.58
SoMe X friendship closeness age 12	0.01	0.02	− 03, 0.05	0.03	0.64	0.26	0.20	− 0.14, 0.65	0.07	0.20
	Social skills at age 16					Time spent with friends at age 16				
		
SoMe X social anxiety age 14	− 0.07	0.04	− 0.15, − 0.004	− 0.06	0.04	− 0.15	0.41	− 0.96, 0.66	− 0.01	0.71
SoMe X friendship closeness age 14	− 0.01	0.03	− 0.06, 0.04	− 0.02	0.70	0.16	0.28	− 0.40, 0.71	0.03	0.58
	Social skills at age 18					Time spent with friends at age 18				
		
SoMe X social anxiety age 16	− 0.24	0.17	− 0.58, 0.10	− 0.18	0.16	0.12	0.57	-1.00, 1.23	0.01	0.84
SoMe X friendship closeness age 16	− 0.08	0.04	− 0.16, − 0.01	− 0.16	0.04	− 0.17	0.39	− 0.93, 0.58	− 0.03	0.66

*Note*. SoMe = Social media use. Between × Within interactions using the between-person component of a social anxiety and friendship closeness as time-varying moderators (Speyer et al., 2023).

## Data Availability

The data that has been used is confidential.
