# Supplementary material for: The new social landscape: Relationships among social media use, social skills, and offline friendships from age 10–18 years
Source: Comput Human Behav. Author manuscript; Available in PMC 2025 Jul 1. (PMC11540422; doi:10.1016/j.chb.2024.108235)
Supplement: Appendix A. Supplementary Data [file NIHMS1985250-supplement-Appendix_A__Supplementary_Data.docx]

**Online material**

**Figure S1****.** Social media use and social skills. Results from the freely estimated Random Intercept Cross-lagged panel model.


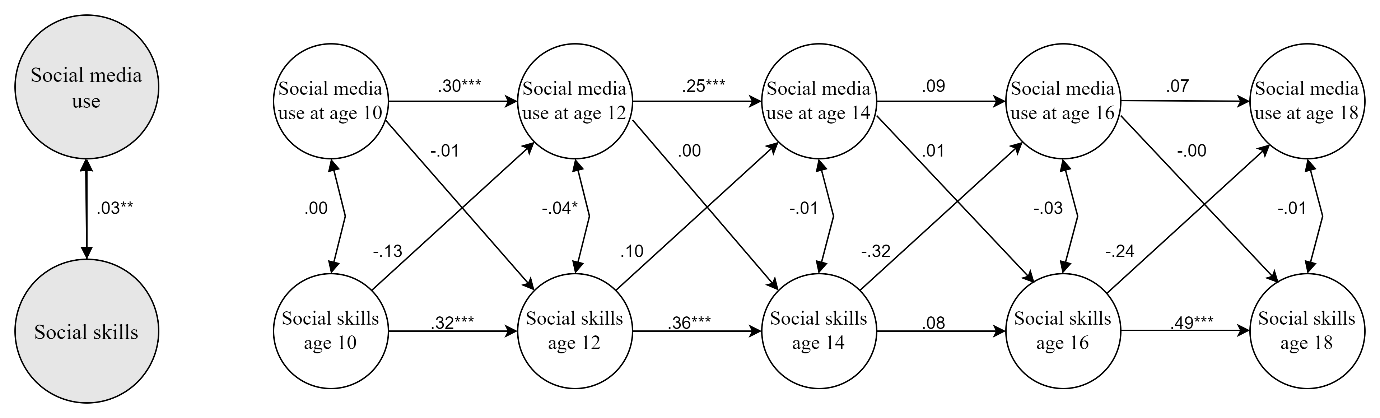


**Figure S2.** Social media use and time spent with offline friends. Results from the freely estimated Random Intercept Cross-lagged panel model.


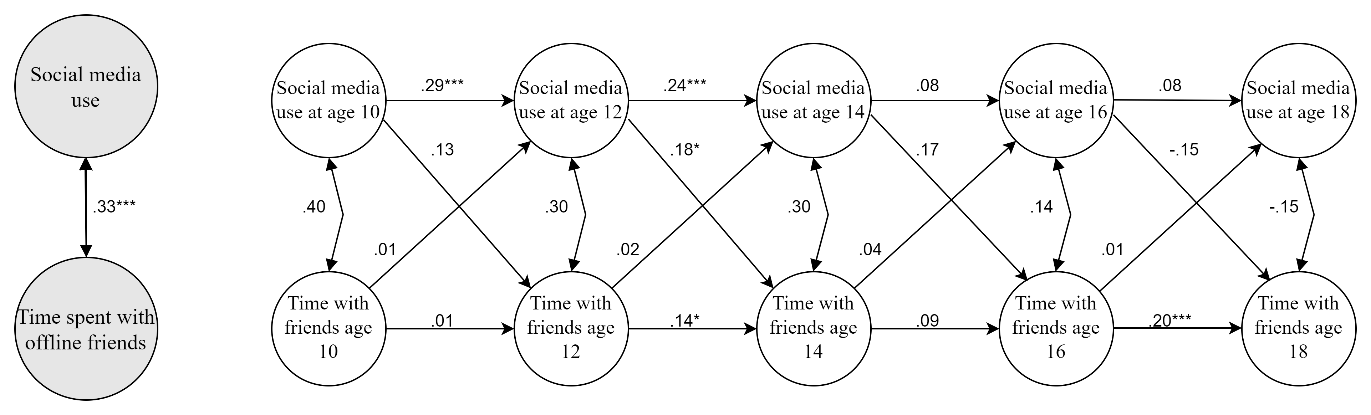


*Note*. The figures display the relations between random intercept estimates (bigger grey circles) and within-person estimates (smaller white circles) of social media use and social skills (Figure 1a), and time spent with offline friends (Figure 1b), respectively (i.e., two separate models). Unstandardized estimates are displayed. For presentation purposes, only the latent variables are displayed, not the observed study variables on which they are based.

**Table S1**. The relation between social media use and social skills, and between social media use and time spent with friends offline. Results from the final estimated Random Intercept Cross-lagged panel models.

|  | Social media use | | | | Social skills | | | | Time spent with friends offline | | | |
| --- | --- | --- | --- | --- | --- | --- | --- | --- | --- | --- | --- | --- |
|  | *B (S.E.)* | 95% CI | *β* | *p* | *B (S.E.)* | 95% CI | *β* | *p* | *B (S.E.)* | 95% CI | *β* | *p* |
|  | Age 12 | | | | | | | | | | | |
| SoMe age 10 |  |  |  |  | -.004 (.01) | -.01, .01 | -.03 | .41 | .12 (.04) | .03, .21 | .09 | .006 |
| SOC age 10 | -.20 (.17) | -.52, .13 | -.03 | .23 |  |  |  |  |  |  |  |  |
| FRI age 10 | .02 (.01) | -.01, .04 | .03 | .12 |  |  |  |  |  |  |  |  |
|  | Age 14 | | | | | | | | | | | |
| SoMe age 12 |  |  |  |  | -.004 (.01) | -.01, .01 | -.03 | .41 | .12 (.04) | .03, .21 | .08 | .006 |
| SOC age 12 | -.20 (.17) | -.52, .13 | -.04 | .23 |  |  |  |  |  |  |  |  |
| FRI age 12 | .02 (.01) | -.01, .04 | .04 | .12 |  |  |  |  |  |  |  |  |
|  | Age 16 | | | | | | | | | | | |
| SoMe age 14 |  |  |  |  | -.004 (.01) | -.01, .01 | -.02 | .41 | .12 (.04) | .03, .21 | .05 | .006 |
| SOC age 14 | -.20 (.17) | -.52, .13 | -.04 | .23 |  |  |  |  |  |  |  |  |
| FRI age 14 | .02 (.01) | -.01, .04 | .04 | .12 |  |  |  |  |  |  |  |  |
|  | Age 18 | | | | | | | | | | | |
| SoMe age 16 |  |  |  |  | -.004 (.01) | -.01, .01 | -.02 | .41 | .12 (.04) | .03, .21 | .05 | .006 |
| SOC age 16 | -.20 (.17) | -.52, .13 | -.06 | .23 |  |  |  |  |  |  |  |  |
| FRI age 16 | .02 (.01) | -.01, .04 | .08 | .12 |  |  |  |  |  |  |  |  |

*Note*. SoMe=social media use; SOC=social skills; FRI=time spent with friends offline.

**Table S2**. Descriptives of moderator variables.

| Variable | Mean | SD |
| --- | --- | --- |
| Social anxiety symptoms age 10 | .03 | .21 |
| Social anxiety symptoms age 12 | .05 | .26 |
| Social anxiety symptoms age 14 | .09 | .34 |
| Social anxiety symptoms age 16 | .44 | .92 |
| Social anxiety symptoms age 18 | - | - |
| Friendship closeness age 10 | 4.27 | .58 |
| Friendship closeness age 12 | 4.40 | .56 |
| Friendship closeness age 14 | 4.39 | .59 |
| Friendship closeness age 16 | 4.42 | .55 |
| Friendship closeness age 18 | 4.39 | .57 |

Note. SoMe = social media use. Age 10-14: Frequency of checking assessed by interview; ages 16-18: Time spent on social media according to the screen time application (see methods for details).

**Sensitivity analyses**

In the sensitivity analyses, the sum of liking, commenting, and posting were replaced by alternative measure of social media use frequency, captured by interview at ages 10, 12, and 14 and objectively measured at ages 16 and 18. In the interview participants were asked about the frequency of checking social media related to specific parts of the day to facilitate correct recall (i.e., number of times in the morning; at school; before noon on weekends; after school and before going to bed; after going to bed), which were summed to constitute the total frequency of checking social media per day (ages 10, 12, 14). At ages 16 and 18 years, following a written procedure, interviewers asked participants to display their screen time use data on their phones, and the interviewer recorded time spent on social media apps (Descriptives are displayed in Table S2). At the time of data collection, this screen time application was only accessible for iPhone users. Objectively measured time spent on social media was thus not assessed in participants using Android, which constituted 18.7% at age 16 and 14% at age 18, respectively.

The same procedure as for the main analyses was used to examine the relationship between this alternative social media measure and social skills and time with friends, respectively. Because the aim was to test whether the findings replicated, we only tested models where cross-lagged paths were constrained to be equal across time, as this was the preferred model of the main analyses. The models showed good fit (Social media use and social skills: χ2=56.27; CFI=.978; TLI=.963; RMSEA= .037 (90%CI: .02, .05); SRMR=.06; Social media use and time spent offline with friends: χ2=49.32; CFI=.959; TLI=.932; RMSEA= .032 (90%CI: .02, .05); SRMR=.05). As for the main results, no relationships between social media use and social skills were found, but youth who increased their social media use over time spent more time with offline friends across ages 10-18 years (Table S3). Also, the sensitivity analyses revealed social media use to be reciprocally related to time spent with offline friends as those who spent more days with friends across the measurement period displayed increased their social media use. In the main analyses, this direction of path was also positive, but non-significant (Figure 1b).

**Table S3**. Descriptives of sensitivity variables.

| Variable | Mean | SD |
| --- | --- | --- |
| SoMe frequency age 10 | 1.54 | 6.30 |
| SoMe frequency age 12 | 7.43 | 15.30 |
| SoMe frequency age 14 | 28.85 | 61.08 |
| SoMe (objectively) age 16 | 3.15 | 1.49 |
| SoMe (objectively) age 18 | 3.58 | 1.61 |

*Note*. SoMe = social media use. Age 10-14: Frequency of checking assessed by interview; ages 16-18: Time spent on social media according to the screen time application (see methods for details).

**Table S4**. Results of RI-CLPM sensitivity analyses

|  | Social media use | | | | Social skills | | | | Time spent with friends offline | | | |
| --- | --- | --- | --- | --- | --- | --- | --- | --- | --- | --- | --- | --- |
|  | *B (S.E.)* | 95% CI | *β* | *p* | *B (S.E.)* | 95% CI | *β* | *p* | *B (S.E.)* | 95% CI | *β* | *p* |
|  | Age 12 | | | | | | | | | | | |
| SoMe age 10 |  |  |  |  | <.001 (<.001) | <.001, <.001 | -.001 | .91 | .42 (.08) | .26, .57 | .25 | <.001 |
| SOC age 10 | -.05 (.37) | -.77, .66 | -.001 | .89 |  |  |  |  |  |  |  |  |
| FRI age 10 | .02 (.01) | .003, .04 | .04 | .02 |  |  |  |  |  |  |  |  |
|  | Age 14 | | | | | | | | | | | |
| SoMe age 12 |  |  |  |  | <.001 (<.001) | <.001, <.001 | -.002 | .91 | .42 (.08) | .26, .57 | .18 | <.001 |
| SOC age 12 | -.05 (.37) | -.77, .66 | <.001 | .89 |  |  |  |  |  |  |  |  |
| FRI age 12 | .02 (.01) | .003, .04 | .04 | .02 |  |  |  |  |  |  |  |  |
|  | Age 16 | | | | | | | | | | | |
| SoMe age 14 |  |  |  |  | <.001 (<.001) | <.001, <.001 | -.01 | .91 | .42 (.08) | .26, .57 | .17 | <.001 |
| SOC age 14 | -.05 (.37) | -.77, .66 | -.01 | .89 |  |  |  |  |  |  |  |  |
| FRI age 14 | .02 (.01) | .003, .04 | .15 | .02 |  |  |  |  |  |  |  |  |
|  | Age 18 | | | | | | | | | | | |
| SoMe age 16 |  |  |  |  | <.001 (<.001) | <.001, <.001 | <.001 | .91 | .42 (.08) | .26, .57 | .05 | <.001 |
| SOC age 16 | -.05 (.37) | -.77, .66 | -.01 | .89 |  |  |  |  |  |  |  |  |
| FRI age 16 | .02 (.01) | .003, .04 | .04 | .02 |  |  |  |  |  |  |  |  |
|  |  |  |  |  |  |  |  |  |  |  |  |  |

*Note*. SoMe=social media use. Age 10-14: Frequency of checking assessed by interview; ages 16-18: Time spent on social media according to the screen time application (see methods for details); SOC=social skills; FRI=time spent with friends offline.
